# A new species and two new records of *Quercus* (Fagaceae) from northern Vietnam

**DOI:** 10.3897/phytokeys.92.21831

**Published:** 2018-01-09

**Authors:** Hoang Thi Binh, Nguyen Van Ngoc, Trinh Ngoc Bon, Shuichiro Tagane, Yoshihisa Suyama, Tetsukazu Yahara

**Affiliations:** 1 Graduate School of Systems Life Sciences, Kyushu University, 744 Motooka, Fukuoka, 819-0395, Japan; 2 Department of Biology, Dalat University, 01 – Phu Dong Thien Vuong, Dalat, Vietnam; 3 Department of Forest Phytodiversity, Silviculture Research Institute, Vietnamese Academy of Forest Sciences, Hanoi, Vietnam; 4 Centre for Asian Conservation Ecology, Kyushu University, 744 Motooka, Fukuoka, 819-0395, Japan; 5 Kawatabi Field Science Center, Graduate School of Agricultural Science, Tohoku University, 232-3 Yomogida, Naruko-onsen, Osaki, Miyagi 989-6711, Japan

**Keywords:** Ba Vi National Park, DNA barcoding, Fagaceae, *Quercus*, Taxonomy, Vietnam, Xuan Lien Nature Reserve

## Abstract

A new species, *Quercus xuanlienensis* Binh, Ngoc & Bon, is described from Xuan Lien Nature Reserve, Vietnam. The new species is morphologically similar to *Q. edithiae* Skan, in having 8–11 pairs of secondary veins, bowl-shaped cupules and ellipsoid to cylindrical-ellipsoid and basally convex nuts. It differs in having serrulate leaf margins only at apical 1/5–1/7, almost entire margins of bracts on cupule and much longer nuts. The species is also similar to *Q. fleuryi* Hickel & A. Camus in having leaves glabrous on both surfaces with only an apically serrulate margin but differs in having shorter petioles, cupules enclosing 1/5 of the nut and much longer nuts. In addition, *Q. disciformis* Chun & Tsiang. and *Q. bella* Chun & Tsiang., previously known from China, are newly recorded from Ba Vi National Park, Vietnam.

## Introduction

*Quercus* L. comprises ca. 400–500 species (Nixon 1993, Valencia-A et al. 2016) and has been divided into two subgenera, Quercus subgenus Cyclobalanopsis (Oerst.) Schneider (ring-cup oaks) characterised by stigma capitate to subcapitate or discoid stigma, cupule bracts being connate or forming concentric or spiral rings and Quercus subgenus Quercus (scale-cup oaks) characterised by usually linear ampliate or broadly ampliated stigma, free and imbricate cupule bracts (Nixon 1993, Manos et al. 1999). In Vietnam, according to Ho (2003) and Ban (2005), a total of 43 *Quercus* species were recorded, amongst which 37 species belong to subgenus Cyclobalanopsis and six species belong to subgenus Quercus. Recently, the following two species were reported and the species of *Quercus* in Vietnam rose to 45 species: *Q. lineata* Blume of subgenus Cyclobalanopsis (Li et al. 2016) and *Q. trungkhanhensis* Binh & Ngoc of subgenus Quercus (Binh et al. in press).

**Figure 1.**
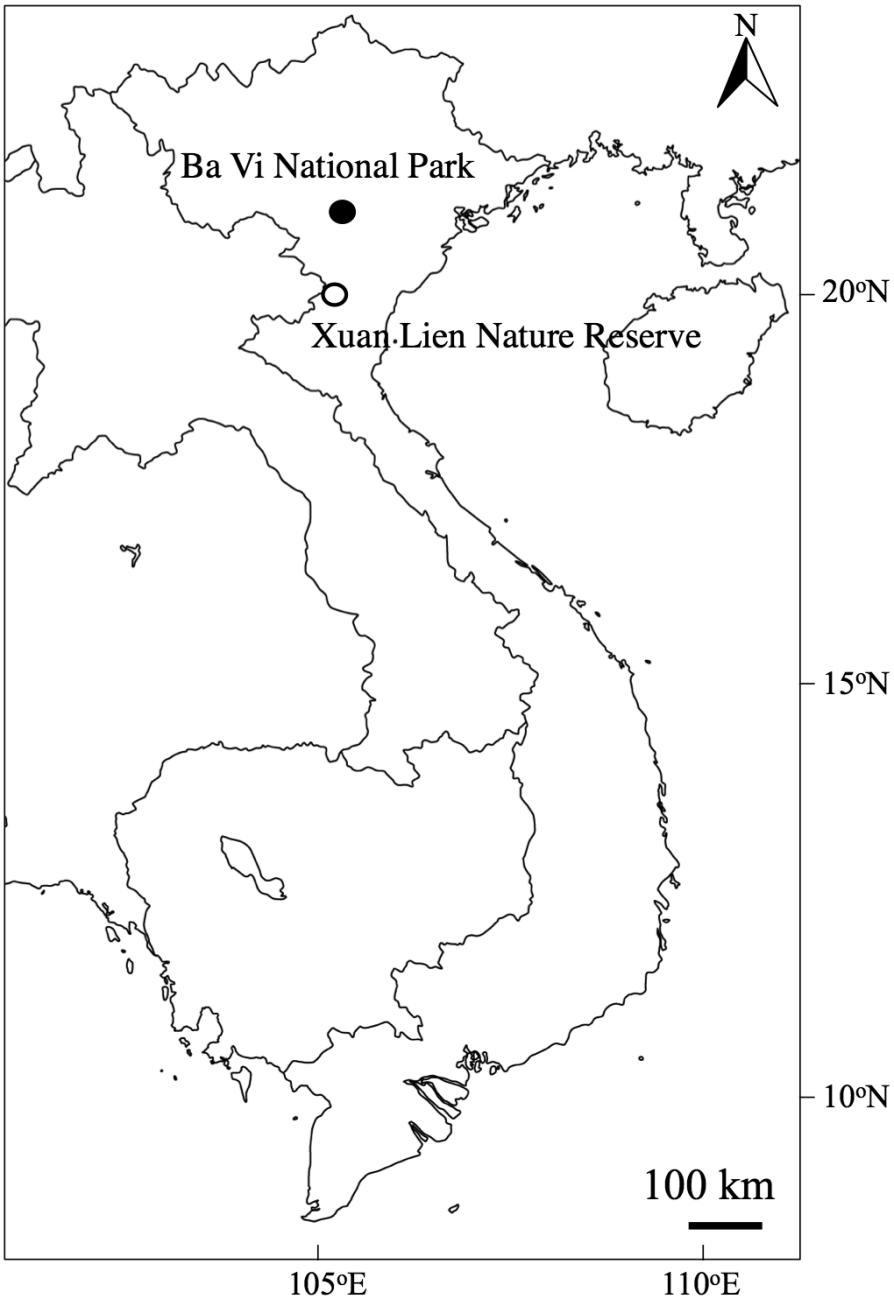
Collection sites of *Quercus xuanlienensis* Binh, Ngoc & Bon, *Quercus disciformis* Chun & Tsiang. and *Quercus bella* Chun & Tsiang.

To widen our knowledge on the Fagaceae of Vietnam, field surveys were undertaken by the authors for 13 conservation areas (national parks, nature reserves and conservation area) in Vietnam and a total of 105 *Quercus* samples were collected. Amongst them, during the field surveys in Xuan Lien Nature Reserve and Ba Vi National Park (Fig. 1), we discovered three unknown species of the subgenus Cyclobalanopsis which were not identical to any of the 38 species of *Cyclobalanopsis* previously recorded from Vietnam (Ho 2003, Ban 2005, Li et al. 2016, Binh et al. in press).

Xuan Lien Nature Reserve, Thuong Xuan District, Thanh Hoa Province, North Central Coast of Vietnam, was established in 1999 with a total area of 21,000 ha. Until now, 1,142 species of vascular plants belonging to 620 genera and 180 families have been recorded (Xuan Lien Nature Reserve 2017). In Fagaceae, 31 species including 17 *Lithocarpus* species (55%), 10 species of *Castanopsis* (32%) and four species of *Quercus* (13%) have been recorded (Xuan Lien Nature Reserve 2017). Ba Vi National Park, Ha Noi Capital, northern Vietnam was established in 1991 with a total area of 7,377 ha (Fig. 1). In this national park, located in the Ba Vi mountain range, 1,201 vascular plant species of 649 genera and 160 families including 19 species of Fagaceae are recorded (Ba Vi National Park 2008).

In this study, a new species is reported from Xuan Lien Nature Reserve and two species are newly recorded from Ba Vi National Park. A new species is described as *Quercus xuanlienensis* Binh, Ngoc & Bon. The two newly recorded species to the country are *Q. disciformis* Chun & Tsiang. and *Q. bella* Chun & Tsiang.

In addition to the morphological examination, DNA sequences and phylogenetic analyses are helpful for delimiting species (Hebert and Gregory 2005, Dick and Webb 2012). Here, DNA sequences of the three species were compared with those of 20 species in Vietnam to confirm that the three species are divergent and thus distinct from the other species. First, two DNA barcode regions were sequenced, the partial genes for the large subunit ribulose-1,5-bisphosphate carboxylase oxygenase (*rbcL*) and maturase K (*matK*) as basic DNA barcodes (CBOL Plant Working Group 2009). However, those sequences show limited divergence in the genus *Quercus* and thus multiple gene markers (Hubert et al. 2014, Simeone et al. 2016), RAD-seq (Cavender-Bares et al. 2015, Fitz-Gibbon et al. 2017) and MIG-seq (Suyama and Matsuki 2015, Binh et al. in review) have been used to determine phylogenetic relationships in *Quercus*. In particular, Binh et al. (in review) successfully used MIG-seq to determine the phylogenetic relationship in the *Quercus langbianensis* complex in Vietnam and revise its taxonomy. In this study, the authors compared the MIG-seq of *Q. xuanlienensis*, *Q. disciformis* and *Q. bella* with those of 18 *Quercus* species studied by Binh et al. (in review) and two additional species *Q. platycalyx* Hickel & A.Camus and *Q. quangtriensis* Hickel & A.Camus that have cupules similar to *Q. disciformis* and *Q. bella*, to determine their identities and phylogenetic relationships.

## Materials and methods

### Morphological observations

The validity of a new species and the identities of newly recorded species were examined based on literature of the genus *Quercus* in Vietnam and its surrounding countries (Camus 1936–1954, Soepadmo 1972, Ho 2003, Huang et al. 1999, Ban 2005, Phengklai 2008, Li et al. 2016, Binh et al. in press), authentic specimens including types by visiting the herbaria DLU, HN, FU, P and VNM and using images available on the web of JSTOR Global Plants (https://plants.jstor.org/) and Chinese Virtual Herbarium (http://www.cvh.org.cn/).

### DNA extraction

DNA was isolated from silica-gel dried leaf materials following the CTAB method (Doyle and Doyle 1987) with minor modifications, as in Toyama et al. (2016). Before the DNA extraction, dry leaf material was milled by QIAGEN TissueLyser to obtain fine powder and the powder was washed up to five times by 1 ml buffer (0.1 M HEPES, pH 8.0; 2% Mercaptoethanol; 1% PVP; 0.05 Ascorbic acid).

### DNA barcoding

DNA regions of the large subunit of ribulose-1,5-bisphosphate carboxylase oxygenase (*rbcL*) and maturase K (*matK*) were amplified and sequenced following the protocols of Kress et al. (2009) and Dunning and Savolainen (2010), respectively.

### Next generation DNA sequencing – MIG-seq

DNA products were used from 105 *Quercus* spp. as templates to amplify thousands of short sequences (loci) from a wide variety of genomes using primers designed for “multiplexed ISSR genotyping by sequencing” (MIG-seq, Suyama and Matsuki 2015). Then presence/absence of each locus in each sample was used for phylogenetic tree reconstruction regardless of whether it has SNP or not. According to the MIG-seq protocol of Suyama and Matsuki (2015) with minor modifications as in Binh et al. (in review), the 1st PCR, multiple non-repetitive regions from various inter-simple-sequence repeat (ISSR) are amplified from genomic DNA by multiplexed PCR with tailed ISSR primers. The 2nd PCR step was performed based on products from the 50 times dilution for each 1st PCR product with deionised water. Then, 3 µl of each 2nd PCR product was pooled as a single mixture library and purified. Subsequently, the Pippin Prep DNA size selection system (Sage Science, Beverly, MA, USA) was used to selected fragments in the size range 350–800 bp. A SYBR green quantitative PCR assay (Library Quantification Kit; Clontech Laboratories, Mountain View, CA, USA) was used to measure the concentration of the size-selected library with approximately 10 pM of libraries. Finally, 10 pM of libraries were used for sequencing on an Illumina MiSeq Sequencer (Illumina, San Diego, CA, USA), using a MiSeq Reagent Kit v3 (150 cycle, Illumina).

### Phylogenetic analyses

In MIG-seq, raw data from 105 samples were pretreated and quality control completed following Suyama and Matsuki (2015) as described in Binh et al. (in review). Subsequently, a list of loci obtained was used for the next steps. This list of loci was detected at least in one individual (1/105=0.01) with the following settings: all samples belong to the same population and threshold frequency of haplotype count in a population (r) = 0.001, a threshold one-order higher than 0.01. Presence/absence (1/0) data of loci were used to compute a distance matrix, construct a neighbour-joining (NJ) tree and the reliability of the tree topology was examined by bootstrapping with 1000 replicates using PHYLYP ver. 3.695 (Shimada and Nishida 2017) as follows; 1000 times re-sampling with Seqboot, distance computation with Restdist, tree construction with NJ and consensus tree construction with Censense. In addition, FigTree v1.4.3 (http://tree.bio.ed.ac.uk/software/figtree/) was used to visualise the resulting tree. A phylogenetic tree for 105 samples including 43 *Quercus* species amongst 44 species previously recorded in Vietnam (data not shown) was constructed and subsequently reduced to 28 samples by focusing on the clades containing *Q. xuanlienensis*, *Q. disciformis*, *Q. bella* and the additional 20 *Quercus* species following Binh et al. (in review). A total of 19,916 loci were used for the final phylogenetic tree.

## Results

### Morphological comparison of a new species and two newly recorded species with similar species

The unknown species (*Quercus xuanlienensis*) collected from Xuan Lien Nature Reserve was not morphologically assignable to any of the species recognised in Vietnam and its surrounding countries. According to Flora of China (Huang et al. 1999) and Illustrated Flora of Vietnam (Ho 2003), *Q. xuanlienensis* is most similar to *Q. edithiae* in leaf size (7–15 × 3–5.8 cm), leaf base (cuneate), petiole length (1.5–2.8 cm long), number of secondary veins (8–11 pairs), cupule shape (bowl-shaped) and nut shape (ellipsoid to cylindrical-ellipsoid). However, *Q. xuanlienensis* is distinct from *Q. edithiae* in having a leaf margin serrated only along its upper 1/5–1/7 (vs. upper 2/3), entire margin of cupule bracts (vs. denticulate except basal 2 or 3 rings) and longer nut (5–6 cm long vs. 4–4.5 cm long) (Table 1). *Quercus xuanlienensis* is also morphologically similar to *Q. fleuryi* Hickel & A. Camus in leaf shape and texture, leaf margin serrulate only at apical 1/5–1/7, entire margin of cupule bracts, basally convex nuts, but *Q. fleuryi* (type: *Fleury 37831*, P [P00753925, P00753926]) showed much larger leaves (14–22 × 5–9 cm) than *Q. xuanlienensis* ((6–)8–11 × 3–4.5 cm)). In addition, *Q. xuanlienensis* is distinct from *Q. fleuryi* in having an ellipsoid bud (vs. ovate), shorter petiole (1.5–2 cm long vs. 2.5–4 cm long), smaller and bowl-shaped cupule, (1.3–1.7 cm high, 1.9–2.1 cm in diam. vs. campanulate to cylindrical, 3.6–3.7 cm high, 3.5 cm in diam.), fewer cupule bracts (7–8 rings vs. 10–13 rings), cupules covering 1/4 to 1/3 of a nut (vs. 2/3) and ellipsoid to cylindrical-ellipsoid (vs. ovoid to cylindrical-ellipsoid) and longer nuts (5–6 cm high, 2–2.3 cm in diam. vs. 3–4.5 cm high, 2–3 cm in diam.) (Table 1).

**Table 1.** Morphological comparison amongst *Quercus xuanlienensis* Binh, Ngoc & Bon, sp. nov., *Quercus edithiae* Skan and *Quercus fleuryi* Hickel & A. Camus.

Characters	*Q. xuanlienensis*	*Q. edithiae* ^(1,2,5)^	*Q. fleuryi* ^(3,4,5)^
Buds shape	Ellipsoid	Ellipsoid to ovoid	Ovoid
Twigs	Tomentose then glabrous	Densely yellowish brown tomentose when young, later glabrous	Densely orange-brown tomentose when young, later glabrous
Stipules	Linear-lanceolate, 10–14 mm long	Caducous, not seen	Caducous, not seen
Leaf margin	Serrate on upper 1/5–1/7 of lamina	Serrate on the upper 2/3 of lamina	Undulate and serrulate on upper 1/6–1/7 of lamina
Leaf surface	Glabrous on both surfaces	Glabrous on upper surface, reddish brown pubescent on lower surface	Glabrous on both surfaces
Leaf base	Cuneate	Cuneate	Broadly cuneate
Leaf size	(6–)8–11(–15) × 3–4.5(–5) cm	7–15 × 3–5.8 cm	14–22 × 5–9 cm
Length of petioles	1.5–2 cm long	1.7–2.8 cm long	2.5–4 cm long
Number of secondary veins	8–11 pairs	9–10 pairs	10–12 pairs
Infructescence	0.8–1 cm long, each infructescence with (1 or) 2 acorns	0.8–1.5 cm long, each infructescence with (2 or) 3 acorns	0.8–1 cm long, each infructescence with (2 or) 3 acorns
Cupule shape and size	Bowl-shaped, 1.3–1.7 cm high, 1.9–2.1 cm in diam.	Bowl-shaped, 1.5–1.7 cm high, 2.3 cm in diam.	Campanulate to cylindric, 3.6–3.7 cm high, 3.5 cm in diam.
Number of rings on cupule	7–8 rings	6–8 rings	10–13 rings
Margin of rings on cupule	Entire	Almost denticulate except basal 2 or 3 which are entire	Entire
Nut enclosure by cupule	Enclosing 1/5 of the nut	Enclosing 1/4 to 1/3 of the nut	Enclosing 2/3 of the nut
Nut shape and size	Ellipsoid to cylindric-ellipsoid, 5–6 cm high, 2–2.3 cm in diam.	Ellipsoid to cylindrical-ellipsoid, 4–4.5 cm high, 2.1 cm in diam.	Ovoid to cylindrical-ellipsoid, 3–4.5 cm high, 2–3 cm in diam.
Base of the nut	Convex, 9–10 mm in diam.	Slightly convex, ca. 7 mm in diam.	Convex, ca. 12 mm in diam.

^(1)^ From the material *Ford 623* (K)

^(2)^ From the original description in Hooker’s Icon. Pl. 27: t. 2661 1901

^(3)^ From the material *Fleury 37831* (P)

^(4)^ From the original description in Bull. Mus. Natl. Hist. Nat. 29: 600 1923

^(5)^ From the description in flora of China (Huang et al. 1999)

According to the key and descriptions in the Flora of China (Huang et al. 1999), the other two unknown taxa from Ba Vi National Park were identified as *Q. disciformis* and *Q. bella*. Excluding slightly thinner leaves and lower teeth, one species is identical with *Q. disciformis* in the following diagnostic characters: leaf blade oblong to obovate-elliptic (6–13 × 2.5–4 cm), margin serrate in the upper 2/3, glabrous on both surfaces when mature; lateral veins 11–13 pairs; petiole ca. 2 cm long; cupule discoid, rim flat when ripe, 3–4 cm in diam., covering base of the nut, scales arranged in 8–10 rings, margin of rings denticulate except apical 2 or 3 entire; nuts oblate 1.5–2 cm high, 2 cm in diam., apex flattened densely appressed hairy. Another species was identified as *Q. bella* having the following characteristics: leaf blade oblong-elliptic to lanceolate (8–15 cm × 2–3.5 cm), base slightly oblique, margin serrate in the upper 1/2; lateral veins 12 pairs of lateral veins; petiole 1–2 cm long; cupule discoid (ca. 0.5 cm × 2.5–3 cm), covering base of the nut, scales arranged in 6–8 rings, margin of rings irregular denticulate; nut oblate nut 1.5–2 cm high and 2.2–3 cm in diam.

### DNA barcoding and MIG-seq

The *rbcL* and *matK* sequences of *Q. xuanlienensis* showed 100% (627/627 bp) and 99% (907/910 bp) homologies with *Q. donnaiensis* and *Q. austrocochinchinensis*, respectively. The *rbcL* and *matK* sequences of *Q. disciformis* and *Q. bella* showed that 100% (696/696 bp) and 100% (833/833 bp) homologies with each other, respectively.

**Figure 2.**
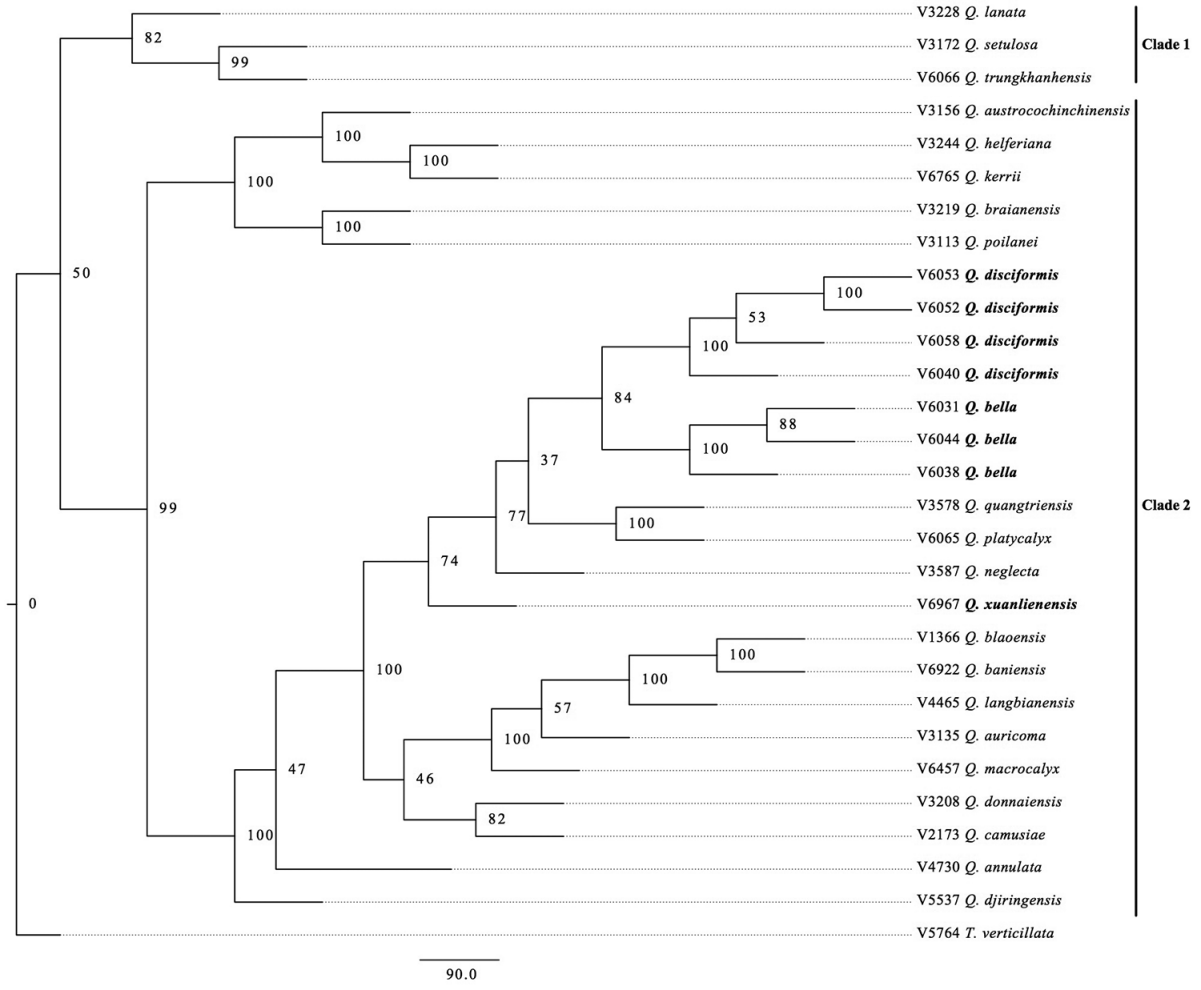
NJ tree of 28 samples of *Quercus* and one *Trigonobalanus* (outgroup) based on presence/absence data of 19,916 MIG-seq loci. Branches are labelled with bootstrap support (% of 1000 replicates).

A phylogenetic tree, inferred using MIG-seq, showed that *Q. xuanlienensis, Q. disciformis* and *Q. bella* are not identical with any of the 20 species from Vietnam. The neighbour-joining (NJ) tree based on MIG-seq data for 28 sample of *Quercus* recognised two major clades using *Trigonobalanus* as an outgroup (Fig. 2). Clade 1 with 82% bootstrap value consists of three species of subgenus Quercus (*Q. lanata*, *Q. setulosa* and *Q. trungkhanhensis*) and Clade 2 with 99% bootstrap value consists of 20 species of subgenus Cyclobalanopsis including *Q. bella, Q. disciformis* and *Q. xuanlienensis*. These three species were clustered with *Q. quangtriensis*, *Q. neglecta* and *Q. platycalyx* and a clade of those six species was strongly supported (74% bootstrap value). Amongst the six species, *Q. xuanlienensis* was separated from the other five species forming a clade with a 74% bootstrap value. Four samples of *Q. disciformis* and three samples of *Q. bella* formed two distinct clades, each supported by 100% bootstrap value. *Quercus disciformis* was sister to *Q. bella* and the clade of those two species had an 84% bootstrap value.

## Discussion

The results of the NJ tree, based on MIG-seq data, showed that *Q. disciformis* is sister to but well differentiated from *Q. bella*. These two species were collected in Ba Vi National Park where they co-occur with similar in leaf and nut morphologies, but differ in the coverage of the cupule (Fig. 4, less than 1/8 in *Q. disciformis* vs. Fig. 5, basal 1/8 to 1/4 in *Q. bella*). According to the Flora of China (Huang et al. 1999), *Q. bella* is recorded from Guangdong, Guangxi and Hainan provinces, whereas *Q. disciformis* is distributed in SW Guangdong, Guangxi, Guizhou, Hainan and Hunan provinces. Ba Vi National Park is located in northern Vietnam, neighbouring Guangxi province and therefore the occurrences of *Q. disciformis* and *Q. bella* there are understandable.

The two species are similar to *Q. platycalyx* and *Q. quangtriensis* in having oblong to oblong-elliptic leaves, usually serrate along leaf margins in the upper 1/2 to 2/3, glabrous on both surfaces when mature, and cupules covering less than 1/3 of the nut and oblate to ovoid nuts (Huang et al. 1999, Phengklai 2008, Ho 2003). The MIG-seq tree showed that those four species are related; the monophyly of a clade including the four species and *Q. neglecta* was supported by a 77% bootstrap value, although the support for the monophyly of the four species is weaker. The affinity of the four species and *Q. neglecta* was unexpected because *Q. neglecta* is an easily distinguished species having linear leaves and small nuts (Huang et al. 1999, Ho 2003).

The MIG-seq tree showed that *Q. xuanlienensis* is related to the above four species and *Q. neglecta* that is morphologically distinct from the other *Quercus* species. From the four species (*Q. disciformis*, *Q. bella*, *Q. quangtriensis* and *Q. platycalyx*), *Q. xuanlienensis* is clearly distinguished by the leaf margin (leaf margin serrulate only at apical 1/5–1/7 in *Q. xuanlienensis* vs. serrate in upper 1/2 to 2/3 in the four species) and nut shape (ellipsoid to cylindrical-ellipsoid vs. oblate to ovoid). Thus, *Q. xuanlienensis* is separated as a distinct species from them.

Whereas *Q. edithiae* is morphologically most similar to *Q. xuanlienensis*, the type specimens of *Q. edithiae* collected from Hong Kong (type: *Ford 623*, K [K000832101, K000832102]) are morphologically distinct from *Q. xuanlienensis* in having distinct serrations, denticulate cupule bracts and smaller nuts and the description of *Q. edithiae* in Flora of China (Huang et al. 1999) agrees with the type specimen. The morphological differences between *Q. edithiae* and *Q. xuanlienensis* are as distinct as those between related species of *Quercus* in Vietnam and its surrounding countries. Huang et al. (1999) recorded *Q. edithiae* in Guangdong, Guangxi, Hainan and Vietnam, but no specimen could be found of *Q. edithiae* collected from Vietnam in any herbarium in Vietnam or on the Chinese Virtual Herbarium website (http://www.cvh.org.cn/). Further studies are needed to confirm the occurrence of *Q. edithiae* itself in Vietnam.

The MIG-seq tree (Fig. 2) was very helpful in deriving the conclusions contained in this paper. As *Q. disciformis* and *Q. bella* are morphologically similar and were collected from the same locality, it was difficult to ascertain whether these are in fact two distinct species and not variants of a single species without the support of the MIG-seq data. Also, the separation of *Q. xuanlienensis* from the other species in Fig. 2 supported the conclusion that it is a new species. The authors also obtained sequences data of *rbcL* and *matK* but the informative content of those sequences was too low to resolve the relationships amongst such closely related species of *Quercus*. Difficulties were faced in determining the sequences of ITS for *Q. disciformis*, *Q. bella* and *Q. xuanlienensis*, most likely due to the low quality of the authors’ samples. MIG-seq is applicable to low quality samples and provides finer resolution of the relationship amongst closely related species (Suyama and Matsuki 2015, Binh et al. in review). Further studies using MIG-seq would be fruitful to elucidate the diversity of *Quercus* in Vietnam, a centre of oak species richness in SE Asia.

## Taxonomic treatments

### Quercus xuanlienensis

Taxon classificationPlantaeFagalesFagaceae

Binh, Ngoc & Bon sp. nov.

urn:lsid:ipni.org:names:77174819-1

Fig. 3

#### Diagnosis.

*Quercus xuanlienensis* is morphologically similar to *Q. edithiae* of China and Vietnam in leaf size (7–15 × 3–5.8 cm), cuneate leaf base, petiole length (1.5–2.8 cm long), number of secondary veins (8–11 pairs), bowl-shaped cupule, ellipsoid to cylindrical-ellipsoid nut and basally convex nut but differs in leaf margin serrulate only at apical 1/5–1/7 (vs. serrate in the upper 2/3), entire bracts of cupule (vs. almost denticulate except basal 2 or 3 rings which is entire), cupule enclosing 1/5 of the nut (vs. enclosing 1/4–1/3 of the nut) and longer nut (5–6 cm long vs. 4–4.5 cm long).

#### Type.

VIETNAM. Thanh Hoa Province, Thuong Xuan District, Xuan Lien Nature Reserve, in evergreen forest around waterfall, alt. 810 m, 19°52'46.7"N, 105°11'34.4"E, 6 Mar. 2017, *Binh HT, Ngoc NV, Bon TN V6967* (holotype KYO!; isotypes DLU!, FU!, P!, VNM!).

**Figure 3.**
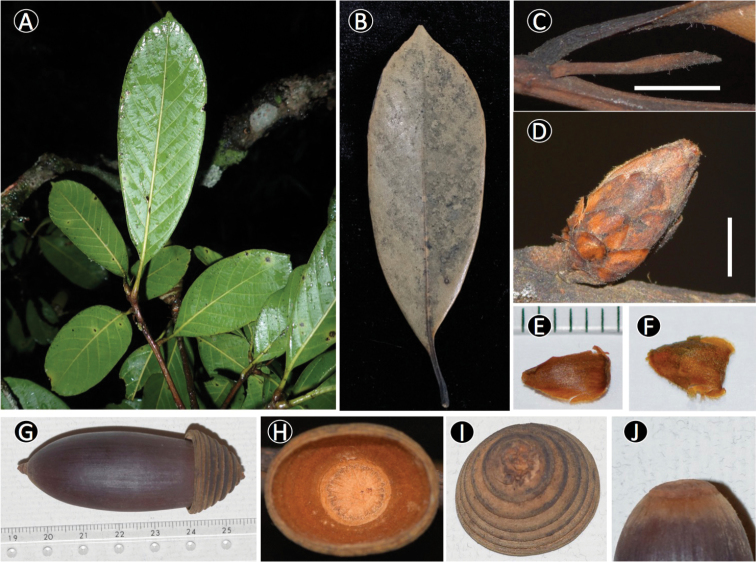
*Quercus xuanlienensis* Binh, Ngoc & Bon. **A** Leafy twig **B** Adaxial side of mature leaf **C** Stipules **D** Bud **E, F** Inside and outside of bud scale **G** Mature fruit **H, I** Inside and outside of cupule **J** Basal scar of the nut. Scale bars: **C** 5 mm **D** 3 mm. Materials from *Binh et al. V6967*.

#### Description.

Tree, ca. 18 m tall. Buds ellipsoid, ca. 9 mm long, ca. 4 mm in diam., scales imbricate, in 4–5 rows, ovate-triangular, ca. 3 × 2.5 mm, apex obtuse, margin ciliate, appressed whitish to yellowish brown hairy on both surfaces. Twigs glabrous when old, lenticellate. Stipules linear-lanceolate, 10–14 mm long, densely appressed hairy, glabrescent outside, glabrous inside. Leaves alternate; blade leathery, oblong-elliptic or obovate, (6–)8–11(–15) × 3–4.5(–5) cm, apex acuminate, acumen up to 0.6 cm long, base cuneate, margin recurved, serrulate in the upper 1/5–1/7, pale brown on the upper surface, yellowish brown to reddish brown on the lower surface when dry, glabrous on both surfaces; midribs ±flat on upper surface, prominent and distinct on lower surface, lateral veins 8–11 pairs, prominent on lower surface, at an angle of 40–45 degrees from midrib, straight and running into the margin, tertiary veins scalariform, indistinct on upper surface, prominent and distinct on lower surface; petioles 1–2 cm long, glabrous. Male and female inflorescences not seen. Infructescences axillary or terminal, erect, rachis 8–10 mm long, 4–5 mm in diam., glabrous, brownish red when fresh, blackish when dried. Fruits 6–6.5 cm high (including cupule), solitary or twin, sessile; cupules bowl–shaped, 1.3–1.7 cm high, 1.9–2.1 cm in diam., enclosing ca. 1/5 of the nut when mature, outside whitish to yellowish brown tomentose to glabrous, inside densely appressed yellowish brown hairy, wall ca. 1–2 mm thick, comprising of bracts, bracts arranged in 7–8 rings, margin of rings entire; nuts ellipsoid to cylindrical-ellipsoid, 5–6 cm high, 2–2.3 cm in diam., apex acute, densely appressed yellowish brown hairy around stylopodia, with stylopodia up to 4 mm long, basal scar 9–10 mm in diam., convex, to 3 mm high, glabrous.

#### Distribution.

Vietnam. Thanh Hoa Province, Thuong Xuan District, Xuan Lien Nature Reserve.

#### Ecology in Vietnam.

At present, only one individual was found in evergreen forest, at 810 m altitude.

#### Etymology.

The specific epithet is derived from the district name of the type locality, Xuan Lien Nature Reserve, Thuong Xuan District, Thanh Hoa Province, North Central Coast of Vietnam.

#### Phenology.

Fruiting specimens were collected in March.

#### GenBank accession no.

Binh et al. V6967: LC331257 (*rbcL*), LC331254 (*matK*).

#### Preliminary conservation status.

*Quercus xuanlienensis* is known for only one individual inside the protected area of Xuan Lien Nature Reserve. According to the criterion D of the IUCN Red List criteria (IUCN 2012), this species is qualified as Critically Endangered (CR).

### Quercus disciformis

Taxon classificationPlantaeFagalesFagaceae

Chun & Tsiang., J. Arnold Arbor. 28: 324 (1947)

Fig. 4

Cyclobalanopsis disciformis (Chun & Tsiang) Y.C. Hsu & H.W. Jen, Acta Bot. Yunnan. 1: 148 (1979).

#### Type.

CHINA. “Hsin-I Hsien, Ling-Tung Pao, Chung-Tung”, 3 Aug. 1931, *C. Wang 31087* (holotype-IBK [catalogue no. IBK00081941, image!], isotype-IBSC [catalogue no. 0117316, image!]).

#### Specimens examined in Vietnam.

Ha Noi, Ba Vi District, Ba Vi National Park, in evergreen forest: alt. 737 m, 21°04'33.88"N, 105°22'03"E, 12 Sept. 2016, *Binh et al. V 6052, V6053, V6058* [fr.] (FU); alt. 1172 m, 21°03'34.1"N, 105°21'54.1"E, 11 Sep. 2016, *Binh et al. V6040* [fr.] (FU).

**Figure 4.**
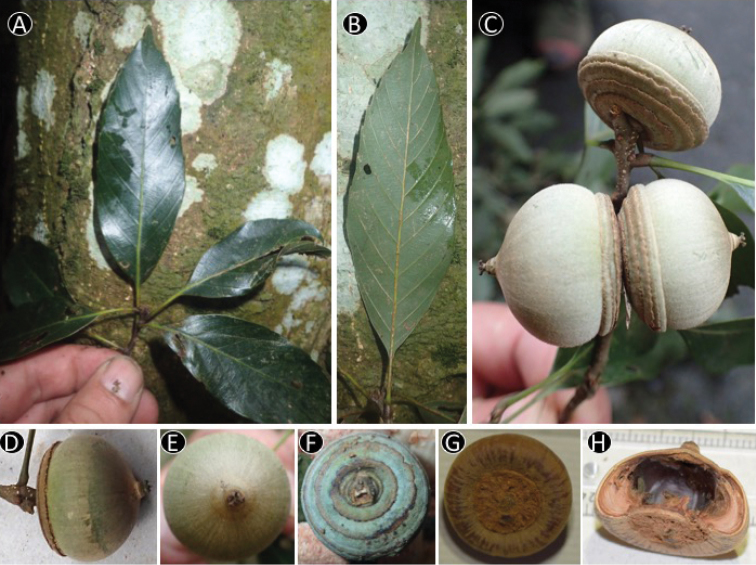
Image of *Quercus disciformis* Chun & Tsiang. from *Binh et al. V6058* (FU) **A** Leafy twig **B** Abaxial side of mature leaf **C–D** Infructescence and mature fruit **E** Nut **F** Cupule **G** Bottom of nut **H** Vertical section of nut.

#### Distribution.

China (Guangdong, Guangxi, Guizhou, Hainan, Hunan) and Vietnam (Ba Vi National Park).

#### Ecology in Vietnam.

In the field survey, only three individuals were found at an altitude of 737 m and one at 1172 m; in evergreen forest.

#### Phenology.

Flowering from March to April, fruiting from August to September in China (Huang 1999). Fruiting in September in Vietnam.

#### GenBank accession no.

*Binh et al. V6058*: LC331258 (*rbcL*), LC331255 (*matK*).

#### Preliminary conservation status.

*Quercus disciformis* is widely distributed from Guizhou to Guangdong and Hainan in China and not recorded as a threatened species in IUCN (2017). The Vietnamese population in Ba Vi National Park extends its distribution range, representing the south western limit. Given the situation, the population in Vietnam is locally important but the category Least Concern (LC) (IUCN 2012, Ban et al. 2007) would be appropriate for this species.

### Quercus bella

Taxon classificationPlantaeFagalesFagaceae

Chun & Tsiang., J. Arnold Arbor. 28: 326 (1947)

Fig. 5

Cyclobalanopsis bella (Chun & Tsiang) Chun ex Y.C. Hsu & H.W. Jen., J. Beijing Forest. Univ. 15(4): 45 (1993).

#### Type.

CHINA. “Fang-Cheng Hsien, Shi-Wan-Ta Shan”, alt. 200–250 m, in sparsely wooded ravine along stream on moist sites, 24 Mar. 1944, *S.H. Chun 4772* (IBSC [catalogue no. 0039624, image!]).

#### Specimens examined in Vietnam.

Ha Noi, Ba Vi District, Ba Vi National Park, in evergreen forest: alt. 600 m, 21°04'40.6"N, 105°22'17.2"E, 11 Sep. 2016, *Binh et al. V 6044, V6038* [fr.] (FU); alt. 703m, 21°04'59.6"N, 105°22'03.6"E, 21 Sep. 2017, *Yahara et. al. V6981* [fr.] (DLU, FU); alt. 1023 m, 21°03'33.7"N, 105°21'39.4"E, 11 Sep. 2016, *Binh et al. V6031* [fr.] (FU).

**Figure 5.**
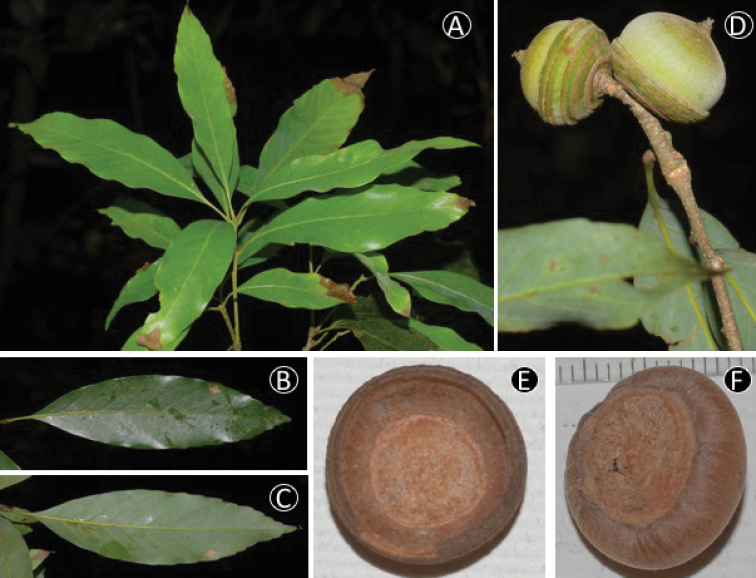
Image of *Quercus bella* Chun & Tsiang. **A** Leafy twig **B** Adaxial side of mature leaf **C** Abaxial side of mature leaf **D** Infructescense and mature fruit (**A–D** from *Yahara et al. V6981* (DLU, FU)) **E** Inside of cupule **F** Bottom of nut (**E–F** from *Binh et al. V6038* (FU))

#### Distribution.

China (Guangdong, Guangxi, Hainan) and Vietnam (Ba Vi National Park, Fig. 1).

#### Ecology in Vietnam.

*Quercus bella* was found on the slopes in evergreen forests in Ba Vi National Park: at alt. 600–1172 m.

#### Phenology.

Flowering from February to April, fruiting from October to December (Huang et al. 1999). Flowering and fruiting specimens were collected from Vietnam in September.

#### GenBank accession no.

*Binh et al. V6038*: LC331259 (*rbcL*), LC331256 (*matK*).

#### Preliminary conservation status.

*Quercus bella* was only previously known as an endemic species to China and distributed in Guangdong, Guangxi and Hainan. The species is not recorded as a threatened species in IUCN (2017). Although only three fruiting individuals of *Q. bella* were collected in Ba Vi National Park, more individuals are expected to occur there and the habitat in the Ba Vi National Park is currently well-protected from anthropogenic activities under the law. Thus, it is appropriate to place this species under the category Least Concern (LC) following IUCN Red List (IUCN 2012) and Vietnam Red Data book (Ban et al. 2007).

## Supplementary Material

XML Treatment for Quercus xuanlienensis

XML Treatment for Quercus disciformis

XML Treatment for Quercus bella
